# Exosome-Derived LncRNA TCONS_00072128 Mediated Osteogenic Differentiation and Inflammation by Caspase 8 Regulation

**DOI:** 10.3389/fgene.2021.831420

**Published:** 2022-03-03

**Authors:** Yongchang Yang, Li Miao, Shuai Chang, Qiuli Zhang, Lijuan Yu, Ping He, Yue Zhang, Weixiao Fan, Jie Liu, Xiaoke Hao

**Affiliations:** ^1^ Institute of Laboratory Medicine Center of Chinese People’s Liberation Army (PLA), Xijing Hospital, Fourth Military Medical University (Air Force Medical University), Xi’an, China; ^2^ Department of Clinical Laboratory, Seventh Medical Center of Chinese PLA General Hospital, Beijing, China; ^3^ Department of Stomatology, Seventh Medical Center of Chinese PLA General Hospital, Beijing, China; ^4^ Department of Blood Transfusion, Seventh Medical Center of Chinese PLA General Hospital, Beijing, China; ^5^ Department of Clinical Laboratory Medicine, Xijing Hospital, Fourth Military Medical University (Air Force Medical University), Xi’an, China; ^6^ BMD Testing Room, Department of Orthopedic, Seventh Medical Center of Chinese PLA General Hospital, Beijing, China; ^7^ Department of Clinical Laboratory, Air Force Hospital in the Northern Theater Command, Shenyang, China; ^8^ College of Medicine, Northwest University, Xi’an, China

**Keywords:** lncRNA, exosome, caspase 8, osteogenic differentiation, PMOP

## Abstract

Postmenopausal osteoporosis (PMOP) is a systemic metabolic bone disease in postmenopausal women. It has been known that long non-coding RNAs (lncRNAs) play a regulatory role in the progression of osteoporosis. However, the mechanism underlying the effects of exosome-derived lncRNA on regulating the occurrence and development of PMOP remains unclear. Exosomes in the serum of patients PMOP were collected and identified. RNA sequencing was performed to obtain the expression profile of exosome-derived lncRNAs in the serum of PMOP patients. RNA sequencing identified 26 differentially expressed lncRNAs from the exosomes between healthy people and PMOP patients. Among them, the expression of TCONS_00072128 was dramatically down-regulated. A co-location method was employed and searched its potential target gene caspase 8. TCONS_00072128 knockdown notably decreased the expression of caspase 8, while the osteogenic differentiation of BMSCs was also reduced. Reversely, TCONS_00072128 overexpression enhanced caspase 8 expression and osteogenic differentiation of BMSCs. Moreover, the continuous expression of caspase 8 regulated by TCONS_00072128 significantly activated inflammation pathways including NLRP3 signaling and NF-κB signaling. Simultaneously, RIPK1 which has emerged as a promising therapeutic target for the treatment of a wide range of human neurodegenerative, autoimmune, and inflammatory diseases, was also phosphorylated. The results of the present study suggested that exosome-derived lncRNA TCONS_00072128 could promote the progression of PMOP by regulating caspase 8. In addition, caspase 8 expression in BMSCs was possible to be a key regulator that balanced cell differentiation and inflammation activation.

## Introduction

Postmenopausal osteoporosis (PMOP) is well known as one of systemic metabolic bone diseases, a decreased estrogen levels *in vivo* is one of the main reasons (Khosla et al., 2012; Khosla and Monroe, 2018). The morbidity among middle-aged older women is up to 50% (Gennari et al., 2016). The pathogenesis of PMOP is based on animbalance between formation of osteoblast and resorption of osteoclasts. This may lead to decreased bone mass, changed the bone tissue structure and increased bone brittleness and fracture (Black and Rosen, 2016). Existing studies have shown that osteoporosis and bone density are heritable, and studies have found that more than 60 susceptibility loci are associated with osteoporosis and bone density. Among them, polymorphisms of several genes include tumor necrosis factor (TNF)-α, interleukin (IL)-10, osteoprotegerin, estrogen receptor 1 gene, estrogen receptor α, cannabinoid receptor 2, Vitamin D receptor gene and low-density lipoprotein receptor-related protein 5 are all related to PMOP (Black and Rosen, 2016; Khosla and Monroe, 2018).

The main clinical treatment of PMOP recently to inhibit bone resorption includes hormone therapy (Gambacciani and Levancini, 2014), calcium supplements (Christenson et al., 2012) and bone marrow mesenchymal stem cells (BMSCs) treatment (Payal et al., 2017). BMSCs, the precursors of osteoblasts, play an important role in tissue regeneration due to their functions of differentiation and self-renewal (Confalonieri et al., 2018). Therefore, it is important in osteoporosis treatment to induce the directional differentiation of BMSCs to bone tissue (Li et al., 2016; Xiao et al., 2016; Liu et al., 2017; Kuang et al., 2019; Nehlin et al., 2019). For this purpose, more studies focus on promoting the osteogenic differentiation capacity of BMSCs by multiple treatment, such as extracellular vesicles and exosomes (Xiao et al., 2017; Liu et al., 2019; Yang et al., 2019; Teng et al., 2020; Li et al., 2021; Tao et al., 2021; Xu et al., 2021). Exosomes coordinate signaling cascades during bone remodeling through non-coding RNA, like miRNA (Xu et al., 2021) or long non-coding RNA (Tao et al., 2021) to regulate RNA transcription. Study have shown that cardiac progenitor cell-derived exosomal miR–21 can decrease oxidative stress to protect myocardium *via* targeting Programmed Cell Death 4 (PDCD4) (Xiao et al., 2016). It was reported that exosomes derived from Wharton’s jelly of human umbilical cord mesenchymal stem cells can transfer miR-21–5p and greatly reduces osteocyte apoptosis for the treatment of glucocorticoid-induced osteonecrosis of femoral heads in rats by activating the AKT serine/threonine kinase (AKT) signaling pathway (Kuang et al., 2019). Liu et al. reported that stem cell-derived exosomes can promote cartilage regeneration (Liu et al., 2017). Li et al. concluded that exosomes derived from human BMSCs can transfer miR-186 to promote osteogenesis in ovariectomy (OVX) rats by the Hippo signaling pathway (Li et al., 2021). However, little is known about the mechanism of PMO, and effective treatment of PMO is limited. It is still to be studied that whether exosome-derived lncRNAs works in osteogenesis. Based on these observations, exploring a new exosome-derived lncRNA associated with PMOP is potential and beneficial to devote a probably therapeutic strategy for PMOP.

In this study, we elucidate the regulatory role and molecular mechanism of TCONS_00072128 /caspase 8 axis in osteogenic differentiation of BMSCs. Our data indicated that TCONS_00072128 has a great effect on osteogenic differentiation and inflammation of BMSCs by regulating caspase 8. The results provided new insights into the regulation of osteogenic differentiation in the treatment of PMOP.

## Materials and Methods

### Sample Collection

Blood samples from PMOP patients and healthy people were collected from the Seventh Medical Center of Chinese PLA General Hospital, Beijing (Table 1). All samples obtained had been informed consent from the patients.

**TABLE 1:** Information of clinical samples.

	PMOP patient		Healthy people	
Number	GS14	GS18	DZh8	DZh13
Sex	female	female	female	female
Age	63	64	57	69
Menopause age	54	48	50	50
Femoral neck T score	−2.4	−2.8	−0.6	−0.7
Femoral neck Z score	−1	−1.3	1.7	1.1
Lumbar spine T score	−2.9	−2.5	1.4	0.7
Lumbar spine Z score	−1.2	−1	2.6	2.8
Height (cm)	156	155	165	164
Weight (kg)	50	55	66	64

For candidates’ enrollment, all candidates are postmenopausal women. They have no Smoke, wine, or bone metabolism drug of histories, with no metabolic syndrome, Skeletal disease, the blood system disease, and cancer. Healthy group had never been diagnosed with osteoporosis or its complications, and the Z value of hip bone density test (BMD) was greater than 1. PMOP patients have similar height and weight ratio, and the Z value of hip bone density test (BMD) was less than −1.

### Cell Culture

MC3T3-E1 subclone 24 cells (CL-0251, China) and dedicated complete medium (CM-0251, China) were purchased from Procell Life Science and Technology Co., Ltd. MC3T3-E1 cells were used for investigation of osteogenic differentiation as a precursor cell model. Cells were cultured in incubator with 5% CO_2_ at 37°C. The medium was changed every 2 days. BMSCs (CP-H166, China) and dedicated complete medium (CM-H116, China) were purchased from Procell Life Science and Technology Co., Ltd. BMSCs were cultured in incubator with 5% CO_2_ at 37°C. The medium was changed every 3 days. When BMSCs were passaged for 3–5 times, cells were treated with lentivirus infection or 5 μM caspase 8 inhibitor Z-IETD-FMK (HY-101297, MCE) for 1 day or 7 days before subsequent experiments. This study was approved by the Seventh Medical Center of Chinese PLA General Hospital.

### Isolation and Characterization of Exosome

The exosome from serum were isolated with exoEasy Maxi Kit (Qiagen, Germany) following manufacturer’s instructions. The size and quantity of isolated exosome were determined with a Nanosight NS300 (NanoSight Ltd., UK), and the morphology of 2% phosphotungstic acid-stained exosome was observed with a transmission electron microscope (H-7650 Hitachi microscope; Hitachi, Japan).

### Exosome RNA Extraction, Library Preparation, and Sequencing

Exosomal RNA was isolated with Total Exosome RNA Isolation reagent (Invitrogen, USA) as manufactures instructed. The RNA concentration was assessed using a NanoDrop 2000 (Thermo Scientific, Waltham, MA, USA). The RNA integrity was analyzed using an Agilent 2,100 Bioanalyze (Agilent Technologies, Foster City, CA, USA). The isolated exosome RNA was incubated with Ribo-Zero rRNA remove beads (Illumina, Inc., San Diego, CA, USA.) to deplete the ribosomal RNA (rRNA) and fragmented into small pieces. The fragmented RNA was ligated to 5’ adapter and then, reverse transcribed with tagged random hexamer, and the cDNAs was ligated to adapter with Unique Molecular Identifiers (UMI). Then, several cycles of PCR amplification were performed to build a library. After the quality inspection of the library, the generated libraries were sequenced on an Illumina HiSeq3000 (Illumina Inc, CA). Subsequently, data analyses were performed *in silico*.

### Sequencing Data Analysis

The raw read sequences were filtered to remove adapter sequences and low‐quality reads by using Trimmomatic software and de-duplicates with UMI. The clean reads were mapped with human reference genome assembly (GRCh38) to define mRNA and lncRNA profiles by HISAT2 aligner. Clean reads were also annotated to GENCODE to calculate reads per kilobase per million reads. Differential expression of lncRNAs was identified by DE Seq package, based on cut-off criteria of log2(FC) >1 or log2(FC) <−1. To annotate gene functions, all differential expressed lncRNAs were aligned against the Gene Ontology (GO) and Kyoto Encyclopedia of Genes (KEGG) database with GO seq R package and KOBAS (v3.0) software.

### Recombinant Lentivirus Infection

As for TCONS_00072128 knockdown, sequence targeting TCONS_00072128 was cloned into Lenti-U6-RFP-Puro vector (#JLSW13252, Gene Line Bioscience). Sequences are listed as below:

Sh-TCONS_00072128 For:

5′-CCA​GAA​CAT​CCT​TCA​CAA​ATT​CAA​GAG​ATT​TGT​GAA​GGA​TGT​TCT​GGT​TTT​TT-3′; LncRNA TCONS_00072128 sequence was cloned into pLent-EF1a-FH-CMV-GP vector (#JLSW13454, Gene Line Bioscience). HEK293T cells were transfected by using second-generation packaging vectors to generate lentiviruses. Then the target cells were infected and selected by puromycin selection.

### Quantitative Real-Time PCR

Target cells were treated with Trizol (#15596026, Invitrogen) according to the reagent instructions to extract the total RNA. The purity and concentration of total RNA were detected by UV spectrophotometer. Total RNA was reverse transcribed into cDNA followed by the requirements of the reverse transcription kit (#K1621, Thermo Scientific). PCR amplification was used by SYBR Green qPCR Master Mix (Thermo Scientific). Subsequently, the expressions of target genes were analyzed after amplification and actin expression as an endogenous reference gene was used. The primers of lncRNA TCONS_00072128 (F: 5- ACA​CCG​CTG​AGA​AGG​ATG​TG -3; R: 5- ACT​CGA​CCA​CGT​AGA​CTC​CA -3), caspase8 (F: 5- ACG​ACC​ATG​AGA​TTG​GCA​GT -3; R: 5- CAG​TCA​CTT​TCA​CCG​GGA​GG-3), RIPK1 (F: 5- GCT​GGC​TGA​GTA​CAC​TGG​AG -3; R: 5- CAG​GGG​TGT​TTA​TCC​CAT​CTG​A-3), NLRP3 (F: 5- ATC​AAC​AGG​CGA​GAC​CTC​TG -3; R: 5- GTC​CTC​CTG​GCA​TAC​CAT​AGA -3), IL-1β(F: 5- GAA​ATG​CCA​CCT​TTT​GAC​AGT​G -3; R: 5- TGG​ATG​CTC​TCA​TCA​GGA​CAG -3), NF-κB (F: 5- CGT​ACA​CGT​CTT​GCC​CTC​AT -3; R: 5- ATA​CCC​CAG​ATC​CTC​CAG​CA -3), NF-κB P65 (F: 5- ATC​ATC​GAA​CAG​CCG​AAG​CA -3; R: 5- TGA​TGG​TGG​GGT​GTG​TCT​TG -3), and Bactin (F: 5- ACC​CTA​AGG​CCA​ACC​GTG​AAA -3; R: 5- ATG​GCG​TGA​GGG​AGA​GCA​TA -3) were designed and synthesized from the Sangon Biotech.

### Western Blot

Western blot was performed to detect exosome-specific biomarkers CD63, CD81 and calnexin. A reducing Laemmli buffer was used to dissolve the target exosomes (5 μg) and boiled for 5 min at 95°C.

Protein samples were resolved in a 10% sodium dodecyl sulfate-polyacrylamide gel (SDS-PAGE). After that, proteins were transferred to a PVDF membrane. The membranes contained proteins were blocked in 5% skimmed milk in PBS containing 0.5% Tween-20 at room temperature for 1 h. When blocking step finished, membranes were probed with the anti-CD63 (#556019, BD Pharmingen), anti-CD81 (#555675, BD Pharmingen) and Calnexin (ab75801, Abcam) at 4°C overnight. Then, membranes were washed with T-TBS 3 times, each time for 5 min. The washed membranes were incubated with the appropriate horseradish peroxidase-labeled secondary antibody (#ab6721, Abcam) for 45 min. The enhanced chemiluminescence (ECL) reagent (#32109, Thermo Scientific) was added on membranes for 1 min to detect the positive immunoreactive bands. Other primary antibodies of Caspase8 (#AF6442,Affinity), RIP (#ab20298, Abcam), Phospho-RIPK1(Ser161) (#66854-1-Ig,Proteintech), NLRP3(#ab263899, Abcam), IL-1 Beta (#66737-1-Ig, Proteintech), NF-kB p65 (#ab32536, Abcam), NF-kB p65 (phospho S536) (#ab76302, Abcam), COL1A1(#GTX112731, GeneTex), Osteopontin (#GTX31886, GeneTex), Osteocaltin (#GTX55255, GeneTex), ALP (#ab108337, Abcam), RUNX2 (#12556S, CST), actin (#ab179467, Abcam) and GAPDH (#ab181602, Abcam) were used to detected protein expressions in BMSCs.

### Alizarin Red Staining & ALP Staining

Alizarin red staining (ARS) was purchased from Sigma (#A5533, Germany). It was performed after exosome treatment for MC3T3-E1 cells, or lentivirus infection infected BMSCs at 1 and 7  days, individually. Cells were fixed with 70% ethanol at room temperature for 60 min. Removed the ethanol and the cells were washed with 1 × PBS (pH7.2, without calcium and magnesium) for 2 times before use. Cells were covered with ARS fluid, avoid light incubation at 37°C for 60 min. cleaned the stained slides slowly with double steaming water for 3–5 min. The images were visualized under a light microscope (Leica DMIRB, Germany).

Alkaline Phosphatase staining (ALP) kit was purchased from Abcam (#ab242287, Germany). The staining process was carried out according to the kit instructions. The MC3T3-E1 cells were treated with exosomes for 14 days. Gently aspirated the medium from the MC3T3-E1 cells and wash with 1 ml of 1× PBST for 2–3 times. Removed the wash solution and added Fixing Solution into the plate, about 0.4 ml per well for a 24-wellplate. Incubated them at room temperature for 2 min. Then removed the Fixing Solution and wash the fixed cells with 1 ml of 1 × PBST twice. Aspirated the washed PBST and added 0.4 ml per well ALP Staining Solution for incubation at room temperature for 30 min. Removed the solution and washed the cells twice. Observed the purple stained cell colonies by using alight microscope.

### Construction of Weighted Correlation Network Analysis Analysis

The WGCNA R software package is a comprehensive collection of R functions for performing various aspects of weighted correlation network analysis. The package includes functions for network construction, module detection, gene selection, calculations of topological properties, data simulation, visualization, and interfacing with external software. Along with the R package we also present R software tutorials. While the methods development was motivated by gene expression data, the underlying data mining approach can be applied to a variety of different settings.

### Statistical Analysis

Statistical analysis was carried out using the SPSS13.0 software (IBM Corp, NY). The significant differences in expression levels between PMOP patients and healthy people groups were tested using a two-tailed Student’s t test. The significance of the GO terms or enrichment of pathway was evaluated using the Fisher’s exact test. *p* values less than 0.05 was considered statistically significant. Unless indicated, results are from at least three-independent experiments.

## Results

### Exosomes Identification

**FIGURE 1:**
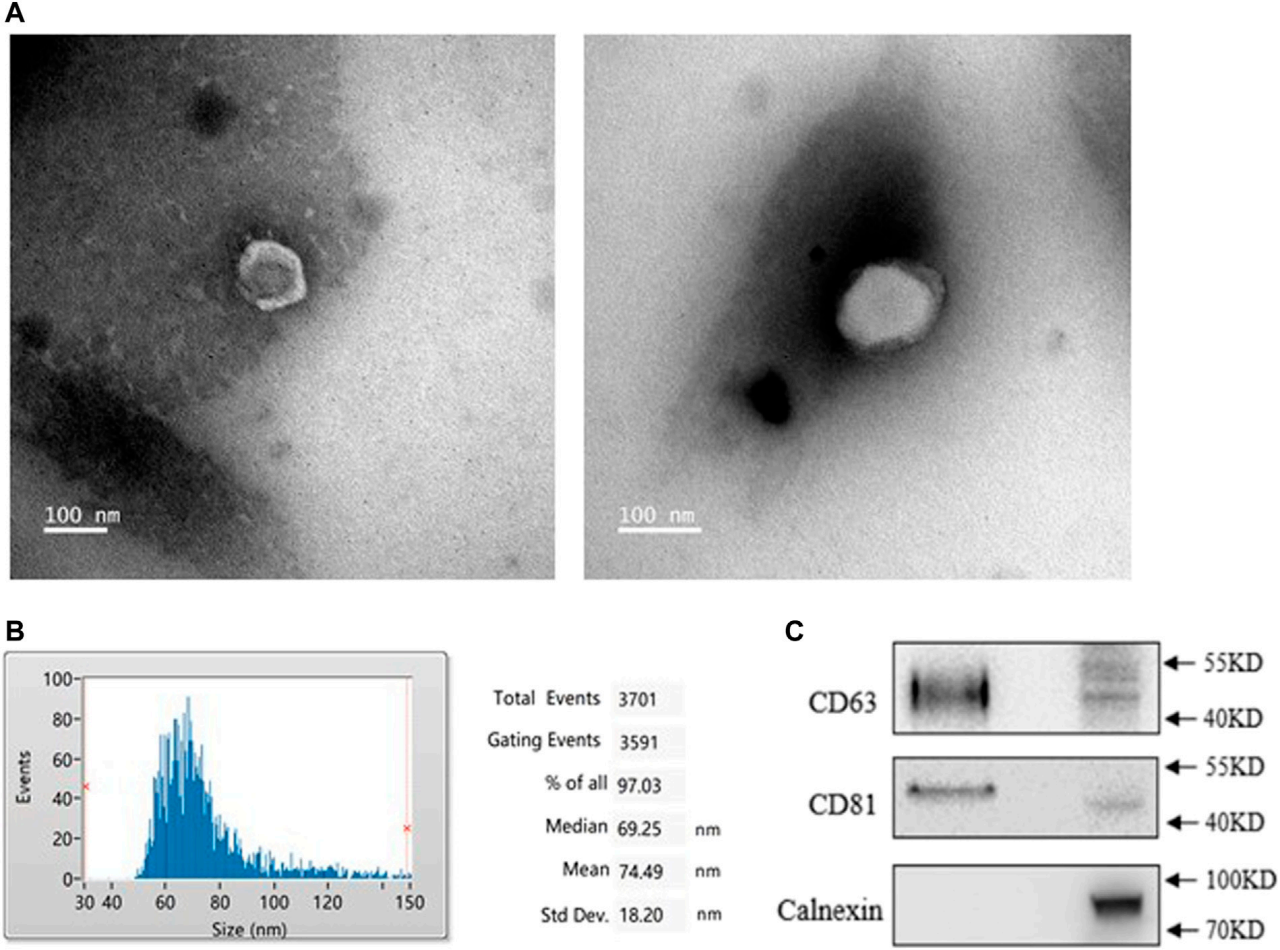
Identification of exosomes from healthy (NC) and PMOP patients (PP). **(A)** Morphology of exosomes was scanned by Transmission electron microscopy (TEM), scale bar = 100 nm. **(B)** The diameter distribution of exosomes. **(C)** Protein expressions of exosomal markersCD63, CD81, and Calnexin. All results are presented with three replicates.

To identify the characteristics of exosomes from PMOP patients, we isolated exosomes from patients’ serum. A typical cup-shaped morphology of exosomes was detected by transmission electron microscopy (TEM) (Figure 1A). Nanoparticle tracking analysis (NTA) showed an average exosome size of 74 ± 18 nm (Figure 1B). CD63 and CD81, the protein markers of exosomes, were detected; in contrast, calnexin was barely detected, which is an integral protein not expressed in exosomes (Figure 1C). These data indicate that the exosomes were successfully isolated with high purity and were suitable for subsequent experiments.

### Roles of Exosomes for Osteogenesis Differentiation

**FIGURE 2:**
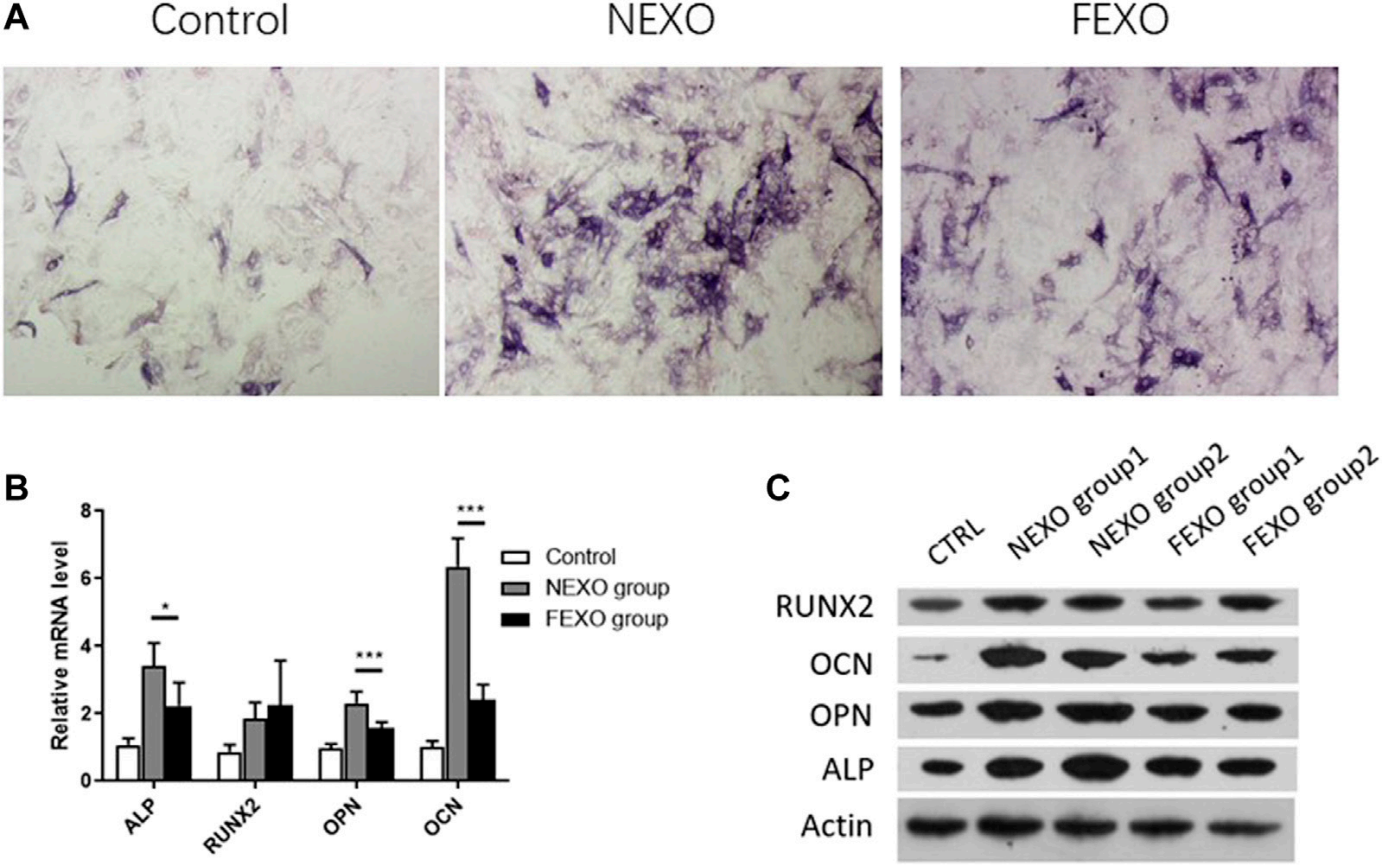
Verification of the effect of exosomes on osteogenic differentiation. **(A)** ALP staining for MC3T3-E1 cells treated with NEXO and FEXO, individually. **(B,C)** qPCR and WB analysis of osteogenesis-related gene expressions in MC3T3-E1 cells. All results are presented with three replicates. *p ≤ 0.05, ***p ≤ 0.001.

Next, we evaluated the osteogenesis differentiation effect on MC3T3-E1 cells by treating exosomes. MC3T3-E1 cells were cultured with osteogenesis induction medium for 14 days. Compared with the treatment of exosomes from healthy people (NEXO group) and PMOP patients (FEXO group), the staining results showed that exosomes from PMOP patients affected ALP expression during MC3T3-E1 cell osteogenesis (Figure 2A). Correspondingly, the expressions of Runx2, OPN, OCN and ALP on both mRNA and protein levels in FEXO group decreased (Figures 2B,C), indicating that exosomes may affect cell osteogenesis differentiation.

### High Throughput Analysis of LncRNAs From Exosomes

**FIGURE 3:**
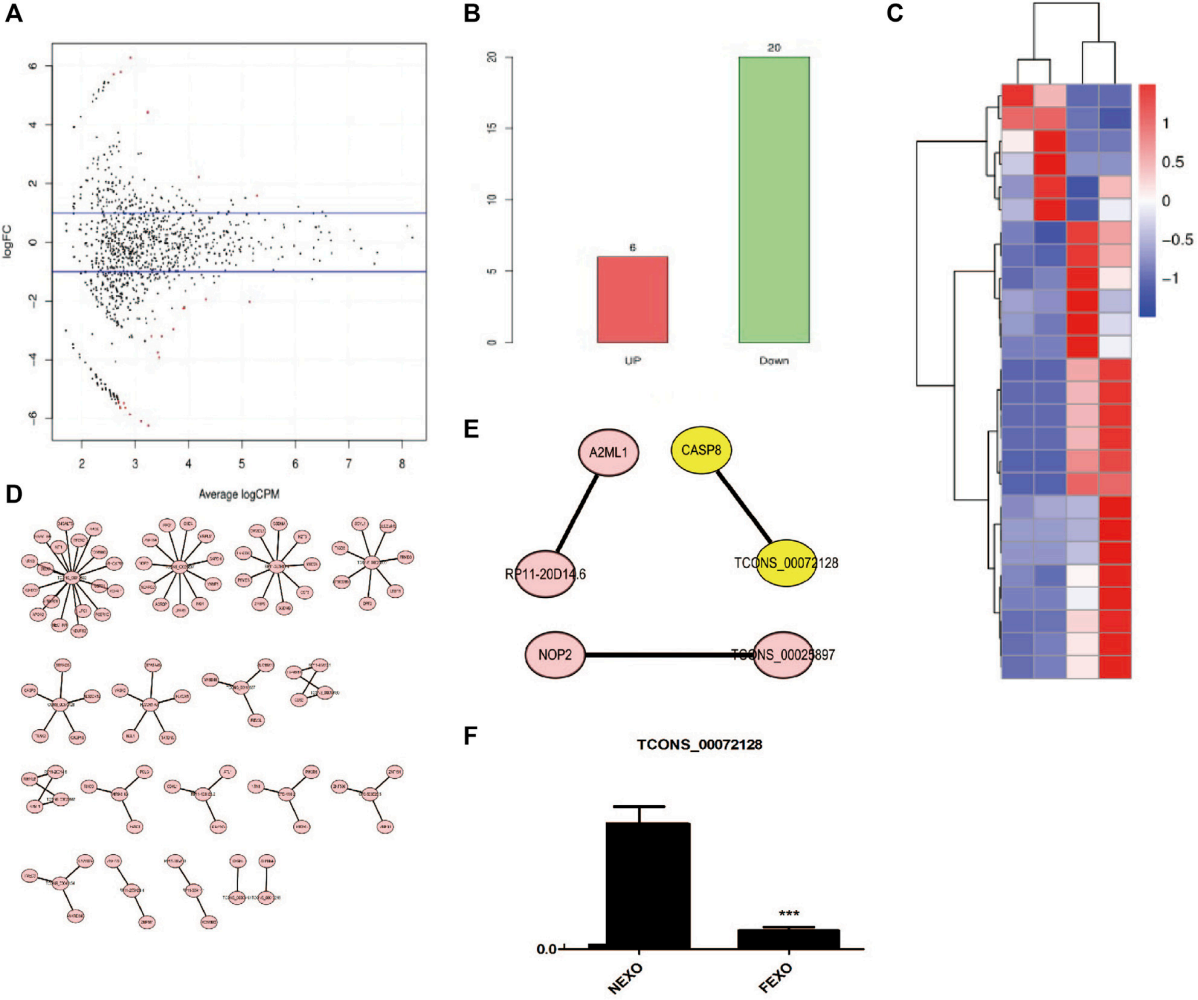
Analysis of differentially expressed lncRNAs in exosomes from healthy (NC) and PMOP patients (PP). **(A)** MA plot of differential expression lncRNAs. **(B)** numbers of differential expression lncRNAs. **(C)** Clustering of differentially expressed genes. Hierarchical clustering based on FPKMs, where log10 (FPKM+1) is used for clustering. **(D)** cis-regulatory target genes of DE lncRNAs were shown. **(E)** Three pairs of potential lncRNAs and mRNAs interactions were both identified in the two methods. **(F)** RNA level of TCONS_00072128 in serum exosomes derived by healthy people and PMOP patients.

There are various lncRNAs in the exosome. When cells take up exosomes, lncRNAs were transferred into cells and subsequently affect the cell function. To distinguish the differences of exosomes from different groups, we performed RNA-seq to explore and identify candidate exosome-derived lncRNAs to evaluate the association of these exosome-derived lncRNAs with PMOP. Total exosomes RNAs from 2 healthy (NC) and 2 PMOP patients (PP) were subjected to RNA-seq. A total of 26 differentially expressed lncRNAs (DE-lncRNAs) were identified, including 20 downregulated lncRNAs and 6 upregulated lncRNAs (Figures 3A–C). Then, the target genes of the DE-lncRNAs were investigated. firstly, the WGCNA (weighted correlation network analysis) was performed to explore the relationship between the DE-lncRNAs and mRNAs. There were 13,572 relationship pairs, including 10,170 pairs of positive correlation and 3,402 pairs of negative correlation (Supplementary Material S1).

Then, the co-location method was employed to search the potential target genes of the DE-lncRNAs. And totally 87 within 100 kb in either direction in the chromosome of DE-lncRNAs were identified as cis-regulatory target genes (Figure 3D). Among the predicted lncRNAs-targets interactions, three pairs were both identified in co-location method and WGCNA (Figure 3E). among them, we focused on a novel defined lncRNA TCONS_00072128and its target gene caspase 8. To verify the RNA level of TCONS_00072128 in exosomes, exosomes derived by patients’ serum were collected to detect RNA level of TCONS_00072128 (Figure 3F). The result was consistent with the prediction of RNA-seq.

### Silencing of TCONS_00072128decreasedcaspase 8 Expression and Osteogenic Differentiation of BMSCs

**FIGURE 4:**
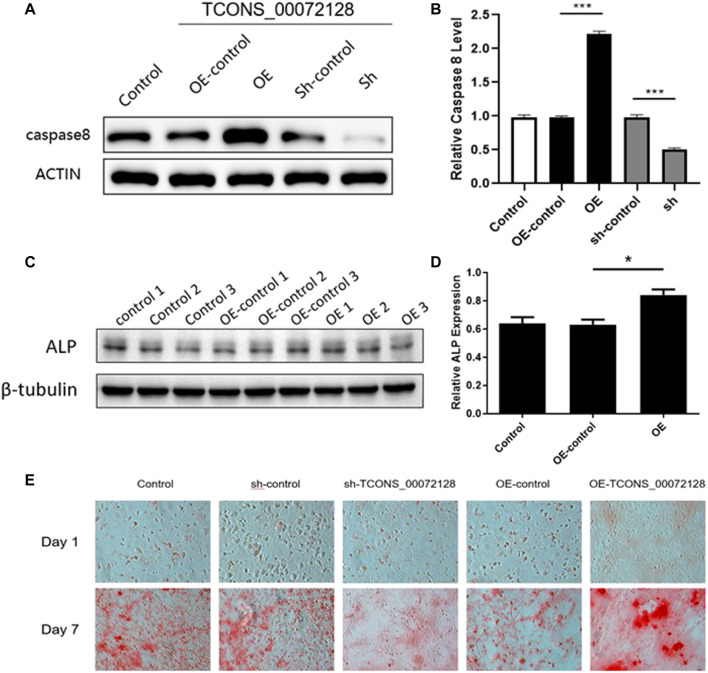
Role of TCONS_00072128-mediated caspase-8 activation in osteogenic differentiation of BMSCs. **(A–D)** Western blot analysis with caspase 8and ALP extracted from BMSCs treated with lentivirus infection. OE, overexpressed TCONS_00072128. Sh, depressed TCONS_00072128. **(E)** ARS staining for BMSCs treated with lentivirus infection at day 1 and day 7. All results are presented with three replicates. *p ≤ 0.05, ***p ≤ 0.001.

Previously studies had revealed that caspase 8 played an important role in osteogenesis differentiation (Meng et al., 2018; Kratochvílová et al., 2020). We then focus on whether TCONS_00072128 affects osteogenesis differentiation. To identify whether TCONS_00072128affects caspase 8, BMSCs were infected with lentivirus sh-TCONS_00072128 and OE-TCONS_00072128. After infections, Cells were induced to differentiate into osteoblasts for 7 days. The results showed that both caspase 8 mRNA expressions and protein levels were significantly suppressed in sh-TCONS_00072128 group (Figures 4A,B). In contrast, OE-TCONS_00072128 group upregulated caspase 8 expression, these data identified the positive correlation between TCONS_00072128 and caspase 8 we predicted by RNA-seq before.

To test whether TCONS_00072128 affect osteogenic differentiation by modulating caspase 8, ALP expression was detected. As shown in Figures 4C,D, OE-TCONS_00072128 group showed higher expression of ALP. This result demonstrated that TCONS_00072128 overexpression positively regulated caspase 8and promoted ALP expression so that heighten the capacity of osteogenic differentiation of BMSCs.

Recent reports referred that caspases-8 participated in osteogenic differentiation (Kratochvílová et al., 2020) and lead to β-catenin proteolysis *in vitro* (Van de Craen et al., 1999). then, we tested whether TCONS_00072128regulated caspase 8 activation affects osteogenesis of BMSCs. The variation of Alizarin red S staining indicated the effect of TCONS_00072128 inosteogenesis (Figure 4E). BMSCs were cultured by using osteogenic differentiation medium for 7 days. Staining for mineral deposition (ARS) confirmed that OE-TCONS_00072128 group promoted BMSCs differentiation over the control at day 7. Reversely, sh-TCONS_00072128 group limited the mineral deposition of BMSCs. These data demonstrated that osteogenic differentiation of BMSCs was occurred in 7 days after induction, these variations were regulated by TCONS_00072128 mediated caspase 8 expression.

### TCONS_00072128-Mediated Caspase 8 Expression Regulates Osteogenic Differentiation and Occurrence of Inflammation

NOD-like receptor family pyrin domain-containing protein 3 (NLRP3) pathway has been known to induce osteogenic differentiation (Wei et al., 2015; Wang et al., 2017). Caspase 8 is one of the impact factors for NLRP3 inflammation (Xu et al., 2020). Different from NLRP3 pathway, NF-κB ligand RANKL contributes to osteoclast formation (Xiong et al., 2018). Therefore, we further explored whether osteogenic differentiation was related by NLRP3 and NF-kB pathways throughTCONS_00072128 mediated caspase 8 expression.

**FIGURE 5:**
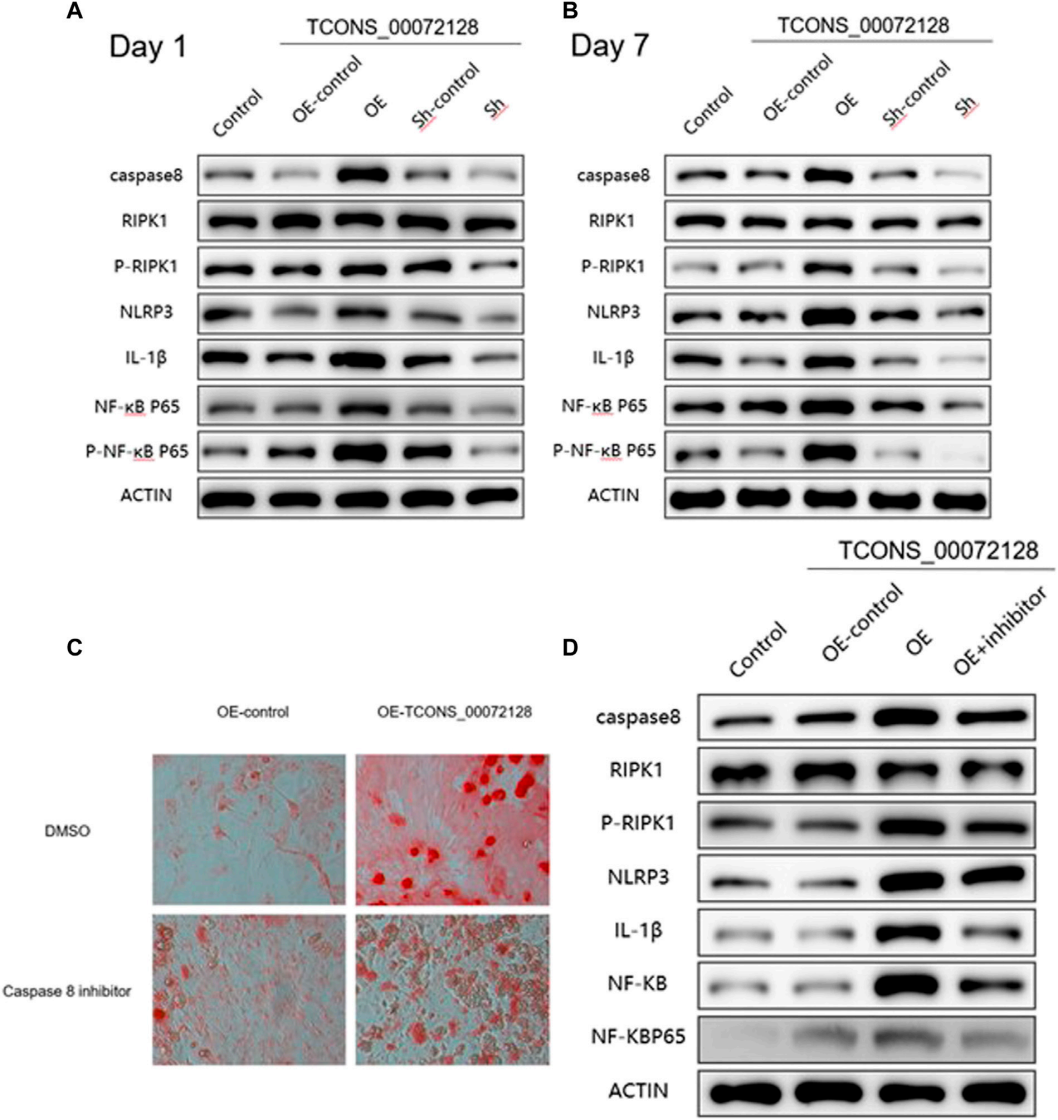
caspase 8 expression regulates osteogenic differentiation and occurrence of inflammation via multiple pathways. **(A–B)** Western blot analysis with caspase 8, RIPK1, p-RIPK1, NLRP3, IL-1β, NF-kB P65 and p-NF-kB P65 extracted from BMSCs treated with lentivirus infection and were induced by osteogenic medium for 1 and 7 days individually. **(C)** ARS staining and **(D)** Western blot analysis for caspase 8 associated proteins extracted from BMSCs treated with lentivirus infection and caspase 8 inhibitor Z-IETD-FMK for 7 days. All results are presented with three replicates. *p ≤ 0.05, ***p ≤ 0.001.

As shown in Figures 5A,B, sh-TCONS_00072128group inhibited caspase 8 expression. Meanwhile, this suppressed expression of caspase 8 decreased NLRP3 and IL-1β expression, as well as RIPK1 and NF-κB p65 phosphorylation at both day 1 and day 7. The former showed consistent results with previous studies, the latter implied the regulatory role of caspase 8 in inflammation development during osteogenic differentiation through NF-κB signaling. During the period of osteogenic differentiation, sh-TCONS_00072128 group, which simulated osteoporosis, inhibited the inflammation factors such as NLRP3and NF-κB, indicating that the inflammation inhibition may have a positive effect; However, the decreased expression of Caspase8 inhibited cell differentiation, even though cell growth was not significantly affected, the differentiation capacity may become lower. On the other hand, although the increased expression of Caspase8 caused by overexpression of TCONS_00072128 improved differentiation capacity, the expression of NLRP3, but not NF-κB P65 phosphorylation, increased significantly during differentiation, especially on day 7.Even more, RIPK1, a key protein for cell apoptosis and necroptosis, its phosphorylation was activated, suggesting that overregulation of caspase8 would activate NLRP3/IL-1β pathways, possibly leading to intensified cell death.

To further clarify the effect of caspase 8 during BMSCs differentiation, we used caspase 8 inhibitor Z-IETD-FMK. Cells were treated with Z-IETD-FMK for 7 days, the staining results indicated that the capacity of BMSCs differentiation was partially inhibited, even the cells had become many more (Figure 5C). Furthermore, the suppressed expression of caspase 8 also decreased NLRP3, IL-1β expression and NF-κB p65 phosphorylation (Figure 5D), which implied thatcaspase8 was possible to balance cell differentiation and activate inflammation induced apoptosis.

## Discussion

Osteoporosis is still a significant medical and socioeconomic challenge around the world, with the characteristics of the systemic impairment of bone mass, and microarchitecture, ultimately enhancing the propensity of fragility fractures (Christenson et al., 2012; Payal et al., 2017; Khosla and Monroe, 2018). Osteoblasts arise from several types of skeletal stem cells, including skeletal, mesenchymal stem cells (MSCs), with osteogenic differentiation potential (Xiao et al., 2016; Nehlin et al., 2019). BMSCs are crucial components in process of new bone formation and are relatively easy to obtain and have a low risk of tumor after implantation. It has been shown that exosomes participate in the regulation of bone homeostasis, which were secreted from BMSCs, osteoclasts, osteoblasts, and endothelial cells. Previous studies have found that osteoclast-secreted exosomes can inhibit osteoblast activity and suppress osteoblastic bone formation (Li et al., 2016; Sun et al., 2016). Exosomes derived from osteoblasts or BMSCs can promote osteoblastogenesis (Cui et al., 2016; Lu et al., 2016). In addition, endothelial cell secreted exosomes can inhibit osteoclastogenesis *in vitro* and reduce bone resorption *in vivo* (Song et al., 2019). According to the functional molecules contained in those exosomes, the molecular mechanism of them was further explored that affecting osteogenic differentiation, such as non-coding RNA (Lu et al., 2016; Fan et al., 2021; Qiu et al., 2021). lncRNAs can participate in epigenetic regulation, transcriptional and posttranscriptional regulation by different mechanisms. lncRNA MEG3 inhibits osteogenic differentiation through down-regulating miR-133a–3p and its expression is increased in bone marrow stem cell of ovariectomized mice and osteoporosis patients (Lu et al., 2016). LncRNA DANCR up-regulated in blood mononuclear cells promoted bone resorption through releasing TNF-α and IL-6 and finally resulted in osteoporosis. LncRNA NTF3-5 promotes osteogenic differentiation and bone regeneration through down-regulating miR-93–3p (Cui et al., 2016; Song et al., 2019). In our study, we focused on a new lncRNA TCONS_00072128 derived from exosomes to verify its regulation effect on osteogenesis. Moreover, exosomes contained TCONS_00072128 are ubiquitous in PMOP patients’ blood, which can be a potential biomarker or direct target to osteogenic differentiation.

The activated NOD-like receptor family pyrin domain-containing protein 3 (NLRP3) inflammasome is an important player in aging-related chronic diseases like osteoporosis, especially because of the causal caspase-1 activation and its correlation to adipose accumulation in bone tissues. NLRP3 inflammasome was reported as the most clinically implicated inflammasome NLRP3 inflammasome is an intracellular protein complex involved in initiation of innate immune response (Wei et al., 2015; Wang et al., 2017). NLRP3 inflammasome contains NLRP3, apoptotic speck protein (ASC) and pro-caspase-1. NLRP3 plays critical roles in multiple chronic diseases. Activated NLRP3 protein is capable of recruiting ASC and pro-caspase-1 to assemble NLRP3 inflammasome. As such, NLRP3 inflammasome sets up the stage for caspase-1 activation, and triggers secretion of inflammatory interleukin (IL)-1β and IL-18 (Wang et al., 2017). Ultimately, NLRP3 inflammasome causes low-grade systemic inflammation and chronic organ failure. The overt increase of cortical bone in NLRP3−/− aging mice compared with wild type counterparts was observed, indicating NLRP3 may twist round MSC differentiation from osteogenesis to adipogenesis(Wei et al., 2015; Wang et al., 2017; Xu et al., 2020).

The relationship between osteogenic differentiation and apoptosis is a dynamic process. Generally, osteogenic differentiation relies on sufficient cells by cell proliferation, so cell apoptosis may not play a major role in the middle and early stages of differentiation, but in late stage. Caspase8 is a member of the apoptotic protein family. It is a molecular switch of apoptosis, necrosis, and pyrolysis (Fritsch et al., 2019). It is highly expressed in osteoclasts (Eva et al., 2018) and is also necessary for the process of osteogenic differentiation (Makio and Akifumi, 2003). Some studies found that TNF-α stimulated human periodontal ligament stem cells inhibited the osteogenic differentiation, in which caspase8 expression is higher (Meng et al., 2018). However, under the treatment of azithromycin, the inhibition of osteogenic differentiation was reversed, as well as the expression of caspase8, indicating the complex relationship between apoptosis and differentiation. In our study, TCONS_00072128 changed the expression of caspase8 accompanied by osteogenic differentiation, even changes in inflammation activation. In recent years, other studies have had similar results. For example, during the process of osteogenic differentiation of MC3T3-E1 cells, the expression intensity of caspase8 increased over time (Kratochvílová et al., 2020). There is a controversial conclusion as to how caspase8 work in the process of osteogenic differentiation. The possible reason may be that caspase8 has non-apoptotic activities, such as promoting cell migration (Dörte et al., 2009) and lymphocyte proliferation (Holli et al., 2008), even more obvious in tumors (Giulia et al., 2018).

Although the expression of caspase 8 promotes differentiation, the continuous accumulation effect leads to activation of the signaling pathways of inflammation and apoptosis, leading to programmed cell death. This is consistent with the results of previous studies (Christina et al., 2015; Kim et al., 2019). Therefore, we infer that in an osteoporotic environment, exosomes with low TCONS_00072128 expression are absorbed by mesenchymal stem cells, thereby reducing the expression of caspase 8. Although mesenchymal stem cells can proliferate as a result, osteogenic differentiation is slow, and even many poorly differentiated, non-differentiated cells are produced, and ultimately cannot undergo osteogenic differentiation. Therefore, in the process of osteogenic differentiation of BMSCs, caspase8 may play multiple roles instead of simply apoptosis. This is also one of our in-depth research directions in the future, to clarify the detailed molecular mechanism of exosome-derived lncRNA regulating caspase 8.

## Data Availability

The data presented in the study are deposited in the BioSample database https://www.ncbi.nlm.nih.gov/biosample/ under Bioproject id - PRJNA808667; accession-SAMN26101027, SAMN26101028, SAMN26101029, SAMN26101030.
